# Is parental coping associated with quality of life in juvenile idiopathic arthritis?

**DOI:** 10.1186/1546-0096-7-7

**Published:** 2009-03-11

**Authors:** Sabrina Cavallo, Debbie Ehrmann Feldman, Bonnie Swaine, Garbis Meshefedjian, Peter N Malleson, Ciarán M Duffy

**Affiliations:** 1 École de Réadaptation, Faculté de Médecine, Université de Montréal and the Montreal Children's Hospital, Montreal, Quebec, Canada; 2 École de Réadaptation, Faculté de Médecine, Université de Montréal, the Montreal Children's Hospital, Groupe de Recherche Interdisciplinaire en Santé and the Public Health Department, Montreal, Quebec, Canada; 3 École de Réadaptation, Faculté de Médecine, Université de Montréal and Centre de Recherche Interdisciplinaire en Réadaptation du Montréal Métropolitain, Institut de Réadaptation de Montréal, Montreal, Quebec, Canada; 4 Public Health Department, Montreal, Quebec, Canada; 5 British Columbia's Children's Hospital and University of British Columbia, Vancouver, British Columbia, Canada; 6 Division of Rheumatology, Department of Paediatrics, Montreal Children's Hospital of the McGill University Health Centre and McGill University, Montreal, Quebec, Canada

## Abstract

Parents of children with a chronic condition such as juvenile arthritis must cope with greater demands than those living with a healthy child. They must adopt different behaviours in order to lessen the impact on the family structure. Parental coping refers to the parent's specific cognitive and behavioural efforts to reduce or manage a demand on the family system. The aims of this study were: to describe coping in a cohort of parents of children with JIA; to determine whether quality of life is associated with parental coping; to explore whether socio-demographic factors such as child's age, family socioeconomic status and family structure are associated with parental coping. One hundred eighty-two parents caring for a child with JIA completed a postal survey at three times over a one-year period, which included the Juvenile Arthritis Quality of Life Questionnaire (JAQQ), the Coping Health Inventory for Parents (CHIP) and questionnaires describing socio-demographic characteristics. Linear mixed models were employed to analyse the association between the child's quality of life and parental coping. Mean total QoL scores (JAQQ) showed that children experienced difficulty in completing specified activities at most just below 25% of the time and results fall off slightly following the 6 month time point. Mean parental coping scores for the CHIP subscales at baseline were 38.4 ± 9.0, 33.4 ± 11.6, 16.5 ± 6.1, for Maintaining Family Integration (maximum score 57), Maintaining Social Support (maximum score 54) and Understanding the Medical Situation (maximum score 24), respectively. Understanding the Medical Situation was deemed most useful. The child's QoL was associated with parental coping. Parents of children with greater psychosocial dysfunction used more coping behaviours related to Understanding the Medical Situation (β coefficient, 0.73; 95% CI, 0.01, 1.45). These findings underscore the importance of helping parents of children with JIA better understand their child's medical situation.

## Background

Juvenile idiopathic arthritis (JIA) is a heterogeneous group of conditions characterized by inflammation of the connective tissues (e.g., the joints) [1]. According to Page it is the fifth most common chronic disease in children [2]. Children affected by JIA report chronic pain, stiffness in joints, fatigue, limitations in mobility which may restrict their participation in daily activities and possibly lead to permanent disability and deformity, and also limiting their quality of life [3,4]. Health related quality of life can be defined as the physical, psychological, and social domains of health, which can be influenced by an individual's experiences, beliefs, expectations and perceptions [5,6]. Quality of life is an important outcome measure used to evaluate the impact of a medical condition such as JIA on the child and the family, as perceived by the patient or by the caregiver [7-9]. These measures must be sensitive to the changes in activities of daily living, fine and gross motor skills, psychosocial integration and physical function associated with the child's development and illness course [10-12]. The limitations highlighted by these measures may in turn affect parents' well-being and ability to cope.

Parents of children with chronic medical conditions such as JIA must cope with greater demands when caring for their child in comparison to those with healthy children [13]. They must adjust their family life to accommodate the frequent medical visits, the multi-component treatment regimen and their child's unpredictable illness course [14,15]. It is important to underline that the child' s quality of life can also influence the way in which a parent cares for their child and may affect the way they cope [16]. Parental coping refers to a specific effort by which the parent attempts to handle or reduce a demand on the family system [17]. According to Folkman et al., coping is defined as the person's constantly changing cognitive and behavioural efforts to manage specific taxing external and/or internal demands [18].

Certain studies have placed great importance on determining what factors related to the child and the parent may influence the child's quality of life [16,19]. However few have examined whether the child's quality of life has any impact on the use of parental coping behaviours. In fact we only found two studies that demonstrated, albeit indirectly, that the child's quality of life and parental coping are associated. The first purports that differences between mothers and fathers coping behaviours regarding information seeking was correlated with a decrease in the quality of life of children diagnosed with cancer [16]. Interestingly the author could not discredit the impact that the child's quality of life might have on parental coping. Another study showed a positive association between better parental coping strategies and greater quality of life in their child one year after a traumatic brain injury [19]. In both these examples the authors are looking to derive the effect of parental coping on the child's quality of life. In our study, we analyse the association between parental coping as the dependent variable and quality of life as one of the independent variables.

We investigated characteristics that may influence parental coping when caring for a child with JIA and the perceived usefulness of family, social and healthcare related resources. Over the years, only a handful of studies have explored the different characteristics of the child, the parent and the family that may impact parental coping. The child's characteristics (e.g. age and gender, quality of life and disease duration), the parent's characteristics (e.g. age, working status) and the family environment characteristics (e.g. family structure, family socioeconomic status) are inter-related and may impact the use of parental coping behaviours [20]. Accordingly, parents may rely on different coping behaviours to answer their needs. We purport that parental perception of the child's quality of life may influence parental coping.

The aims of this study were 1) to describe the child's quality of life as perceived by their parents and parental coping behaviours in a cohort of parents of children with JIA, 2) to determine whether the child's quality of life is associated with parental coping, 3) to explore what socio-demographic factors are associated with parental coping. As an additional objective, we examined whether the child's quality of life and socio-demographic factors are associated with parental distress. By identifying coping behaviours and the usefulness that parents attribute to them, we can guide parents in finding appropriate services for respite and emotional support in an attempt to provide quality care for their child with JIA, as well as their family.

## Methods

The data collection was carried out between September 2000 and January 2004.

### Study population

Parents, either the mother or the father, caring for a child with JIA (n = 235) who attended the JIA clinic at two Canadian pediatric hospitals (Montreal Children's Hospital-McGill University Health Centre, n = 144 and British Columbia's Children's Hospital in Vancouver, n = 91) agreed to participate. Parents were eligible for the study if they spoke and understood either English or French.

### Data collection

This study was a secondary analysis of data collected as part of a larger study examining adherence to treatment for children with JIA. Parents of children with JIA were asked to complete the following questionnaires and to return them by mail: 1) the Juvenile Arthritis Quality of Life Questionnaire (JAQQ), 2) the Coping Health Inventory for Parents (CHIP), 3) the Symptom Checklist-90-Revised (SCL-90-R), 4) a socio-demographic questionnaire. Data were collected at three time points through self-report questionnaires: entry into the study (baseline), six months and twelve months later.

The study was approved by the Montreal Children's Hospital and the British Columbia's Children's Hospital Institutional Review Boards.

### Measures

The Juvenile Arthritis Quality of life Questionnaire (JAQQ), a valid and responsive tool was used to measure disease-specific quality of life in children with JIA [21-23]. This questionnaire has four domains: (1) gross motor function (17 items), (2) fine motor function (16 items), (3) psychosocial function (22 items), (4) systemic symptoms (19 items) and a section, not included in the total score assessing pain using a 100 mm visual analogue scale. Each domain is scored using a seven-point Likert scale from never i.e. 0% (1) to always i.e. 100% (7) and a zero score if the item is not applicable to the child; higher scores correspond to a greater dysfunction (i.e. lower quality of life) due to the effects of arthritis or its treatment within the past two weeks. The total JAQQ score is obtained by computing the mean of the four subscales mean scores [23]. Content validity is supported by the correlations between the different subscales of the JAQQ and measures of joint disease activity and pain, ranging from r = 0.32 to r = 0.49 [23]. Good correlations were found between all the JAQQ subscales, pain and the physician's global evaluation of change, demonstrating that the JAQQ is responsive to important change in child's functional status [23,24]. Internal consistency and test-retest reliability were not reported.

The Coping Health Inventory for Parents (CHIP) was used to measure parental coping patterns [25]. This 45-item questionnaire is a valid and reliable measure by which parents rate their perception of how useful certain coping behaviours are by way of a four-point Likert scale from not helpful (0) to extremely helpful (3). These coping behaviours are grouped into three patterns. The first coping pattern is Maintaining family integration, cooperation, and an optimistic definition of the situation (19 items, maximum score = 57), which refers to, for example parents participating in activities with other family members or getting other family members to help with chores and tasks at home. The second coping pattern is Maintaining social support, self esteem, and psychological stability (18 items, maximum score = 54), which refers to, for example parents getting away from the home care tasks and responsibilities for some relief or talking to someone about how they feel. The third coping pattern is Understanding the medical situation through communication and consultation with healthcare professionals (8 items, maximum score = 24), which refers to, for example parents talking with healthcare professionals (nurse, physician, occupational therapist, physiotherapist, social worker, etc.) concerning their child's condition. The higher the score the more useful the particular type of coping pattern. For the purpose of this study, we derived what we have coined the percentage maximum score for each pattern to allow for better comparison of coping results across the study period. This percentage was calculated by dividing the total score for each coping pattern by the maximal possible score of that specific pattern. The internal consistency of this tool is good with Cronbach alphas of 0.79; 0.79; 0.71 for each pattern, respectively [25]. The CHIP has fair concurrent validity and correlates with the Family Empowerment Scales [25].

The Symptom Checklist-90-Revised (SCL-90-R) was used to evaluate parental distress [26,27]. This is a 90 item self-report tool measuring nine symptom clusters by using a five-point rating scale of distress ranging from not at all (0) to extremely (4). We used Derogatis' measure of "caseness" where a Global Severity Index (GSI) t-score equal to or greater than 63 is indicative of clinical psychological distress [27]. The SCL-90-R demonstrates adequate internal scale consistency from 0.77 to 0.90 and reasonably good test-retest reliability (one week) from 0.78 to 0.90 [27]. Validity of the SCL90R was supported by significant associations with the corresponding DSM-IIIR/DSM-IV symptom disorders [28].

Socio-demographic and other characteristics were collected by a questionnaire developed specifically for this study. It included questions about the parent's employment status, parent's age and family socioeconomic status. In lieu of household income maternal education was used as a proxy indicator of the family's socioeconomic status. Other information such as child's age, gender and disease duration (in years) was abstracted from the medical chart.

### Analysis

In our cohort we enhanced the sample power by taking advantage of the repetitive aspect of the study and included all time intervals in our analysis using linear mixed modeling.

Univariate analyses were conducted to describe baseline characteristics of the sample and to examine distributions of different variables. These characteristics were also used to compare participants and non-participants at baseline, 6-month and 12-month intervals. Mean and standard deviation of coping patterns were also calculated at these three time periods. We performed multivariate analyses using Linear Mixed Models to determine associations between the child's quality of life and each of 1) parental coping and 2) parental psychological distress, adjusting for relevant socio-demographic characteristics of interest and those mentioned in the literature review: child's age, gender and duration of the disease; parent's age and, parent's employment status; family socioeconomic status and family structure. We used two models to illustrate each of the above two research questions: in the first model total JAQQ score was the main independent variable, while in the second model four subscales of the JAQQ were the main independent variables. In our analysis, we used Linear Mixed Modeling, which is a generalization of the standard linear model that allows to combine same subjects' data over the study period in one analysis. Furthermore, we used residual maximum likelihood as the method of parameter estimation with compound symmetry as the covariance structure based on Schwarz's Bayesian Information Criterion [29]. All variables were treated as fixed-effect parameters except for the intercept, which was treated as a random-effect parameter.

Analyses were performed using SPSS software version 14 (Chicago, Il) [30].

## Results

Of the initial 235 parents who consented to participate, 182 (77.4%) returned questionnaires: 120 from Montreal and 62 from Vancouver. There were no significant differences between participants and non-participants at baseline on several socio-demographic variables such as child's age, gender and duration of disease, parent's age, family's socioeconomic status, parent's work status and family structure. However, the mean active joint count (ajc) was higher in children of participants (1.8 versus 0.6; p = 0.001) compared to those of non-participants. At six months there were 136 participants, respondents differed from non-respondents with respect to the child's quality of life. Namely, non-respondents presented with poorer quality of life than respondents (data not shown). Finally, at twelve months there were 115 participants. At this time point a significantly greater number of non-participant children were older (11.4 versus 9.5 years) and with a longer duration of disease (5.0 versus 3.8 years) than participants.

Of the 182 parents who participated in our study each had only one child with JIA. Mothers had a mean age of 39.6 (6.0) years. Only 25 parents (15.7%) presented with clinical psychological distress at baseline (Table 1). There were 182 children with JIA in our sample, 69.2% were females. At baseline, the means (SD) for child's age and disease duration were 10.2 (4.4) years (range 2.0–18.0 years) and 4.2 (3.6) years (range 0.1–15.6 years), respectively. The percentage of children with each JIA classification was 44.5% oligoarthritis, 20.3% polyarthritis, 9.3% systemic arthritis, 9.9% enthesitis related arthritis, 10.4% psoriatic arthritis, and 5.5% other. Mean total JAQQ scores and subscale scores are presented at baseline, 6 months and 12 months (Table 2). Mean total QoL scores show that children experienced difficulty in completing specified activities at most just under 25% of the time. These results also show that the child's quality of life falls off slightly following the six-month follow-up and remains relatively stable after that time interval. Mean scores for the CHIP at baseline, six and twelve month follow-up are presented in Table 3. At baseline, Understanding the Medical Situation was found to be slightly more useful than Maintaining Family Integration, while Maintaining Social Support was found to be the least useful coping pattern. Maximum score for each of these coping patterns were respectively 24, 57 and 54. Mean scores for each coping pattern remained relatively stable over the study period.

**Table 1:** Baseline characteristics of parents and children from the JIA study sample (n = 182).

**Parents characteristics**	
	Mean (SD)

**Parent's age (yrs)**	
Mothers	39.6 (6.0)
Fathers	42.2 (6.6)

	n *(%)

**Parent's employment status**	
Working	106 (69.3)

**Parental psychological distress**	
< 63	134 (84.3)
≥ 63 (high distress)	25 (15.7)

**Family characteristics**	

	n* (%)

**Family structure**	
One parent family	46 (26.6)
Two parent family	127 (73.4)

**Family socioeconomic status**	
Low	60 (39.2)

**Child's characteristics**	

	Mean (SD)

**Child's age (yrs)**	10.2 (4.4) range 2.0–18.0

**Duration of Disease (yrs)**	4.2 (3.6) range 0.1–15.6

**Pain score (VAS)**	16.9 (23.1)

**Active joint count (ajc)**	1.8 (0.5)

	n *(%)

**Child's gender**	
Female	126 (69.2)

*excluding missing cases

**Table 2:** Mean (SD) Juvenile Arthritis Quality of life Questionnaire (JAQQ) scores at baseline, 6-month and 12-month study period.

	Baseline (n = 181)	6-month (n = 120)	12-month (n = 104)
Gross Motor	2.6 (1.8)	2.0 (1.5)	2.0 (1.5)

Fine Motor	1.6 (1.2)	1.2 (0.8)	1.2 (1.1)

Psychosocial	2.2 (1.3)	1.8 (1.4)	1.9 (1.2)

Systemic Symptoms	2.6 (1.3)	2.3 (1.5)	2.3 (1.4)

Total JAQQ score	2.2 (1.2)	1.7 (1.1)	1.8 (1.0)

**Table 3:** Mean (SD) Coping Health Inventory (CHIP) scores of parents at baseline, 6-month and 12-month study period.

	Baseline (n = 162)		6-month (n = 101)		12-month (n = 98)	
	
	Total pattern score (SD)	% of maximum score	Total pattern score (SD)	% of maximum score	Total pattern score (SD)	% of maximum score
Maintaining Family Integration (max: 57)	38.4 (9.0)	67.4	37.4 (9.2)	65.6	36.3 (10.4)	63.7

Maintaining Social Support (max: 54)	33.4 (11.6)	61.9	33.8 (11.0)	62.6	33.4 (12.6)	61.9

Understanding the Medical Situation (max: 24)	16.5 (6.1)	68.8	16.3 (5.7)	67.9	15.1 (6.5)	62.9

Table 4 presents the three parental coping patterns against the total quality of life score (total JAQQ). Age and gender adjusted results indicate an inverse significant relationship between total JAQQ score and Maintaining Social Support coping pattern. Namely, a higher total JAQQ score was significantly associated with a decrease in the perceived usefulness of this coping pattern (β coefficient, -1.86; 95% CI, -3.16, -0.56). On the other hand, family's socioeconomic status was also significantly associated with certain parental coping patterns. For instance, lower socioeconomic status was associated with more perceived usefulness of Maintaining Family Integration (β coefficient, 3.94; 95% CI, 0.75, 7.12) and Understanding the Medical Situation (β coefficient, 3.39; 95% CI, 1.44, 5.34).

**Table 4:** Association between quality of life (JAQQ total score) and coping (family integration, social support and medical situation) based on results of Linear Mixed Model Analysis.

	Model 1a^‡^	Model 1b^‡^	Model 1c^‡^
	
	Maintaining Family Integration	Maintaining Social Support	Understanding Medical Situation
	β (95% CI)*	β (95% CI)*	β (95% CI)*
Total JAQQ score	-0.78 (-1.80, 0.23)	-1.86 (-3.16, -0.56)**	0.08 (-0.65, 0.81)

Family socioeconomic status: (low versus high)	3.94 (0.75, 7.12)**	3.15 (-0.59, 6.88)	3.39 (1.44, 5.34)**

^‡ ^These models are adjusted for age and gender variables of the child but their β (95% CI) are not shown.

* β (95% CI) = β coefficient and 95% confidence interval.

** p-value < 0.05

With respect to the second model (Table 5), and among the four subscales of the JAQQ, psychosocial and systemic symptoms were only significantly related to the coping pattern related to Understanding the Medical Situation. Mainly, families whose children had greater psychosocial dysfunction tended to find most useful behaviours classified as Understanding the Medical Situation (β coefficient, 0.73; 95% CI, 0.01, 1.45). Also, greater frequency of systemic symptoms, were associated with decreased perceived usefulness of the same coping pattern (β coefficient, -0.81; 95% CI, -1.55, -0.06). The family's socioeconomic status manifested associations similar to that of models in Table 4.

**Table 5:** Association between quality of life (four subscales of the JAQQ) and coping (family integration, social support and medical situation) based on results of Linear Mixed Model Analysis.

	Model 2a^‡^	Model 2b^‡^	Model 2c^‡^
	
	Maintaining Family Integration	Maintaining Social Support	Understanding Medical Situation
	β (95% CI)*	β (95% CI)*	β (95% CI)*
Gross motor	-0.24 (-1.10, 0.61)	-0.78 (-1.90, 0.33)	0.35 (-0.27, 0.97)

Fine motor	-0.17 (-1.20, 0.87)	-0.03 (-1.40, 1.33)	-0.38 (-1.16, 0.39)

Psychosocial	0.29 (-0.68, 1.26)	-0.18 (-1.45, 1.10)	0.73 (0.01, 1.45)**

Systemic symptoms	-0.69 (-1.72, 0.34)	-0.80 (-2.13, 0.52)	-0.81 (-1.55, -0.06)**

Family socioeconomic status: (low versus high)	4.13 (0.93, 7.32)**	3.45 (-0.32, 7.22)	3.32 (1.37, 5.27)**

^‡ ^These models are adjusted for age and gender variables of the child but their β (95% CI) are not shown.

* β (95% CI) = β coefficient and 95% confidence interval.

** p-value < 0.05

Table 6 summarizes the association of the child's quality of life as perceived by the parent with parental psychological distress. These models are also adjusted for child's age and gender. Results reveal that a higher score for child psychosocial difficulties was significantly associated (β coefficient, 0.39; 95% CI, 0.05, 0.73) with greater parental psychological distress.

**Table 6:** Association between total quality of life JAQQ score (model 1) or: four subscales of the JAQQ quality of life score (model 2) and parental distress based on results of Linear Mixed Model Analysis.

	Model 1^‡^	Model 2^‡^
	
	Parental distress (t-score GSI)	Parental distress (t-score GSI)
	β (95% CI)*	β (95% CI)*
Total JAQQ score	0.14 (-0.24, 0.52)	N/A

Four subscales of the JAQQ	N/A	
Gross Motor		0.09 (-0.20, 0.39)
Fine Motor		-0.20 (-0.63, 0.23)
Psychosocial		0.39 (0.05, 0.73)**
Systemic symptoms		-0.07 (-0.38, 0.23)

^‡ ^These models are adjusted for age and gender variables of the child but their β (95% CI) are not shown.

* β (95% CI) = β coefficient and 95% confidence interval.

** p-value < 0.05

## Discussion

Parents of children who had more psychosocial dysfunction found that understanding the medical situation through consultation with healthcare professionals was the most useful coping pattern. Furthermore, greater psychosocial dysfunction in children with JIA appears to be associated with a higher degree of parental psychological distress. On the other hand lower quality of life i.e. greater overall child dysfunction, as shown by higher total JAQQ scores and more frequent systemic symptoms, were significantly related to less perceived usefulness of certain parental coping patterns.

Parents whose children had more psychosocial difficulties found coping behaviours related to Understanding the Medical Situation, which includes communicating and consulting with healthcare professionals most useful. Poor psychosocial function in children with juvenile rheumatic disease may impact parental depression and may increase emotional strain [31]. To help alleviate some of this strain parents may seek information to enhance their understanding of their child's medical situation in an attempt to improve their child's health and possibly improve their child's social integration. In comparison, parents whose children had more frequent systemic symptoms did not find it useful to use coping behaviours related to the pattern Understanding the Medical Situation. These parents may be so overwhelmed by their child's disease and its devastating effect on their child's quality of life, or possibly so well informed about the limitations of the medical interventions, that they do not find communicating with healthcare professionals useful.

A higher total JAQQ score was significantly associated with less perceived usefulness of coping behaviours related to Maintaining Social Support, indicating that the poorer the child's overall quality of life, the less useful, parents find such behaviours as seeking out social services and participating in non-professional support groups. Parents may feel overwhelmed requiring more external social support than is available in order to cope with their family situation [32].

Our findings did not show a significant association between child's age and parental coping, partly corroborating those of Tak et al. who found no significant association between child's age and maternal coping among parents of children diagnosed with congenital heart disease [33]. However, McCubbin reported that mothers rely less on seeking social support in older children than in younger children with cystic fibrosis [34]. Our study has shown that parents of lower socioeconomic status seem to find the coping patterns of Maintaining Family Integration and Understanding the Medical Situation useful, which entails completing activities with family members and asking doctors and other healthcare professionals about their child's disease. These parents are possibly more dependent on external support given to them by family members and on information provided by healthcare professionals.

Although child's overall quality of life (total JAQQ score) was not related to parental distress, we did find that higher child psychosocial dysfunction was associated with higher parental distress. Lustig et al. reported that greater dysfunction among children with juvenile rheumatoid arthritis (JRA) is associated with more psychological distress among mothers [35]. Psychosocial dysfunction affects a child's interactions with parents, siblings, teachers and peers. These children may present with decreased participation in school and extra-curricular activities. Parents may feel more psychological distress in response to their child's limited social integration. The child's functional status and the parent's level of psychological distress may influence parents' choice of preferred coping behaviours.

### Study limitations

There are a number of limitations to these findings. The child's quality of life falls off slightly following the six-month follow-up. If those who dropped out of the study were also less inclined to use certain coping patterns, then this may have affected our results. Information on parental coping was based purely on parents' self-report of the usefulness of specified coping patterns. We were not able to examine whether the parents truly used the coping patterns or whether they are effective. Although, we adjusted for age, gender, and related socio-demographic characteristics, there may be other factors that we did not measure that could potentially influence a child's quality of life and parental coping (e.g. parental and family dynamics, stress, the presence of other siblings either ill or healthy, the child's level of distress).

## Conclusion

Parents who perceived a lower quality of life for their child (specifically in terms of psychosocial dysfunction) tended to seek out medical information from healthcare professionals, possibly in an attempt to better their child's situation. Understanding the Medical Situation is a coping pattern that was deemed most useful by the parents in this study. These findings may support the clinical implication of healthcare professionals as important sources of medical information for parents. On the other hand, parents of children who had more frequent systemic symptoms relied less on the information given to them about their child's condition. This may be due to the lack of successful communication between healthcare professionals and parents of children with JIA who are not doing well. These findings may support the need for clinicians to adopt effective techniques to help parents better understand their children's medical situation.

## Abbreviations

AJC: Active joint count; CHIP: Coping health inventory for parents; GSI: Global Severity Index; QoL: Quality of life; SES: Socio-economic status; SPSS: Statistical Package for the Social Sciences; SCL-90-R: Symptom Checklist- 90- revised.

## Competing interests

The authors declare that they have no competing interests.

## Authors' contributions

SC drafted the manuscript and completed part of the data analysis. DF helped with planning and conception of the study and analysis, and helped with writing and revising the manuscript. BS helped with writing and revising the manuscript. GM completed data analysis and helped with revising the manuscript. PM and CD both helped with revising the manuscript.
